# Extra! Extracellular Effector Delivery into Host Cells via the Type 3 Secretion System

**DOI:** 10.1128/mBio.00594-17

**Published:** 2017-05-02

**Authors:** Melissa M. Kendall

**Affiliations:** Department of Microbiology, Immunology, and Cancer Biology, University of Virginia School of Medicine, Charlottesville, Virginia, USA

**Keywords:** EPEC, effectors, secretion systems

## Abstract

The type three secretion system (T3SS) is critical for the virulence of diverse bacterial pathogens. Pathogens use the T3SS to deliver effector proteins into host cells and manipulate host signaling pathways. The prevailing mechanism is that effectors translocate from inside the T3SS directly into the host cell. Recent studies reveal an alternative mechanism of effector translocation, in which an effector protein located outside the bacterial cell relies on the T3SS for delivery into host cells. Tejeda-Dominguez et al. (F. Tejeda-Dominguez, J. Huerta-Cantillo, L. Chavez-Dueñas, and F. Navarro-Garcia, mBio 8:e00184-17, 2017, https://doi.org/10.1128/mBio.00184-17) demonstrate that the EspC effector of enteropathogenic *Escherichia coli* is translocated by binding to the outside of the T3SS and subsequently gains access to the host cell cytoplasm through the T3SS pore embedded within the host cell membrane. This work reveals a novel mechanism of translocation that is likely relevant for a variety of other pathogens that use the T3SS as part of their virulence arsenal.

## COMMENTARY

**FIG 1:**
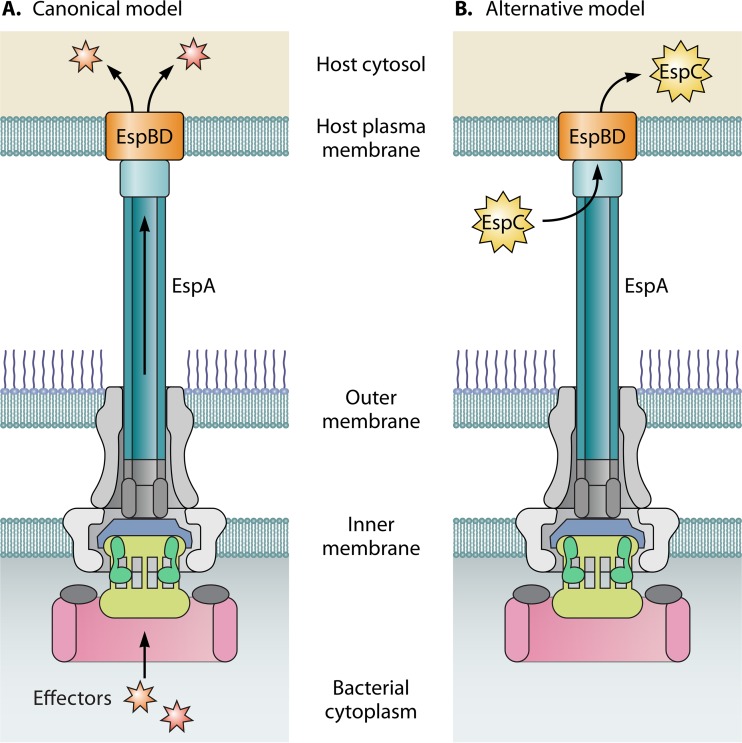
(A) Canonical model of T3SS-mediated effector delivery. Effector proteins located in the bacterial cytosol translocate through the inside of the T3SS for delivery into the host cell. (B) Alternative mechanism of T3SS-dependent effector delivery. Extracellular proteins, including EspA, interact with the outside of the T3SS needle filament, EspA, to gain access to the translocon pore (EspB-EspD) and subsequent entry into the host cell.

The type three secretion system (T3SS) is a major bacterial virulence factor associated with many Gram-negative pathogens that infect humans, animals, insects, and plants (1). The T3SS enables bacteria to deliver or translocate effector proteins into host cells, and these effectors alter diverse host processes, including cellular trafficking, barrier dysfunction, and immune responses. The T3SS system is comprised generally of three main parts, the basal body, needle, and translocon (2). The proteins that form the basal body are anchored in the bacterial inner and outer cell membranes and form a socket-like structure comprised of several rings and a center rod (3). The needle structure protrudes from the basal body into the extracellular space and is associated with a tip or filament protein that interacts directly with the host cell. The translocon proteins insert into the host cell membrane and form a pore, through which bacterial proteins gain entry into host cells. Together, these components form a hollow tube that connects the bacterial cell to the host cell (Fig. 1A). The long-standing model of T3SS-dependent effector delivery is that effectors located in the bacterial cytosol are translocated directly into the host cell through this “molecular syringe” in a one-step process. Significantly, emerging data support an alternative model in which extracytoplasmic proteins are also translocated into host cells in a T3SS-dependent manner (4, 5).

Now, in a recent article, Tejeda-Dominguez et al. provide mechanistic insights into T3SS-mediated translocation of an extracellular protein, EspC, of enteropathogenic *Escherichia coli* (EPEC) (6). EspC belongs to the SPATE (serine protease autotransporters of *Enterobacteriaceae*) family, a group of serine protease autotransporters conserved within the *Enterobacteriaceae* family. EspC contributes to the virulence of EPEC by cleaving host proteins, including hemoglobin, pepsin, and focal adhesion proteins—the latter of which requires T3SS-dependent host cell internalization (5, 7–9). EspC secretion from the bacterial cell is mediated by the type 5 secretion system (T5SS) or autotransporter system but requires the T3SS for host cell internalization. It was previously shown that EspC interacts with the EPEC T3SS needle filament protein EspA (5), which suggested a potential mechanism for EspC translocation. To investigate this idea, Tejeda-Dominguez et al. (6) divided the EspC passenger domain into three segments (containing the amino terminus, middle region, or carboxy terminus) and purified each component. The authors first demonstrated that the middle portion of the EspC passenger domain was required both for strong interaction with EspA and translocation into host cells. Further analyses revealed that 21 amino acids within the middle domain were highly conserved with the translocation sequence of YopH, a *Yersinia pseudotuberculosis* cell surface protein that also requires the T3SS for translocation (4). These conserved amino acid residues play a specific role in EspC translocation, as the authors provided evidence that an EspC protein fragment lacking the 19 amino acids was still able to bind EspA but could not be detected in the cytoplasm of epithelial cells.

Because EspC interacted with EspA through a specific binding motif that is different than the EspC translocation sequence, the authors (6) hypothesized that EspC interacts with additional components of the T3SS to gain entrance into the host cell. The proteins EspB and EspD comprise the EPEC T3SS translocon. Although EspB is required for EspC translocation, EspB and EspC do not directly interact (5). Similarly, EspA does not interact with EspB, but it does interact with EspD (10). Therefore, the authors examined EspC interaction with the EspA-EspD complex. Using a combination of affinity chromatography and surface plasma resonance experiments, Tejeda-Dominguez et al. (6) demonstrated that EspC binds the EspA-EspD complex with greater affinity than to EspA alone, which is consistent with previous work that showed that EspC preferentially targets EspA-EspD structures compared to EspA (11). Interestingly, the authors also reported higher disassociation kinetics of EspC from the EspA-EspD complex compared to EspA alone. A recent study showed that EspC promotes degradation of EspA and EspD to control pore formation (11). Although not tested, EspC-mediated degradation of EspA and EspD may contribute to the increased dissociation kinetics of the EspC-EspA-EspD complex, which is necessary for EspC translocation into the host cell.

To further examine EspC interaction with EspA and EspD, the authors (6) performed confocal microscopy analyses to visualize EspC interaction with EspA and EspD. In these experiments, EspC was visualized along the EspA filaments, whereas EspC-EspD interactions were observed as punctate features. On the basis of these collective findings, the authors proposed a model in which EspC interaction with the tip of the EspA filament connects EspC to the translocon pore to gain entry into host cells.

The authors (6) predicted that if their model was correct, steric hindrance of the translocon pore should prevent EspC translocation. To test this idea, the authors (6) infected epithelial cells with EPEC (containing a deletion of *espC*) and then added anti-EspB or anti-EspD antibodies to block pore access, followed by the addition of purified EspC protein. EspC could not be detected in the cytoplasm of epithelial cells treated with the anti-EspB/anti-EspD antibodies; however, EspC was detected when an unrelated antibody was used. To confirm these results, the authors performed an elegant study using confocal microscopy to examine EspC translocation during EPEC infection of epithelial cells with or without sterically blocking the translocon pore. After treatment with anti-EspB/anti-EspD antibodies, exogenously added EspC was localized to bacterial microcolonies and is restricted to outside the epithelial cells. In contrast, after treatment with a control antibody, exogenous EspC could be observed visualized within epithelial cells. These findings provide proof of principle that the extracellular effector EspC is delivered into host cells through the translocon pore, revealing a novel mechanism of T3SS-dependent effector translocation.

Despite the considerable progress made in understanding the biogenesis and structure of bacterial T3SSs as well as the regulation of T3SS and function of secreted effectors, the mechanism of effector delivery has been less clear. The prevailing model of the T3SS-dependent effector delivery occurs through a one-step mechanism in which effectors travel from the bacterial cytoplasm directly into the host cell (Fig. 1A); however, definitive evidence of this process has only recently been reported (12). Now, new findings demonstrate an alternative mechanism for delivery of effectors that interact with the outside of the T3SS to gain access to the host cell cytosol (Fig. 1B). Significantly, the authors identified a conserved translocation motif in EPEC and *Y. pseudotuberculosis*, which suggests a shared mechanism for T3SS-dependent delivery of extracellular proteins (6). Future investigations are necessary to fully understand the extent and types of extracellular proteins that rely on the T3SS for translocation. Additionally, studies that examine the mechanism of pore translocation by extracellular proteins (i.e., determining whether these proteins require folding and/or unfolding steps to traverse the translocon pore) as well as how the timing of extracellular protein translocation is coordinated with translocation of cytosolic effectors are warranted. Nevertheless, the study by Tejeda-Dominguez et al. (6) reveal exciting new insights into bacterial physiology, pathogenesis, and host-bacterium interactions.
